# Treating Complex Trauma in Adolescence: A Case Study of Brief Focal Psychotherapy After Vicarious Gender Violence and Child Abuse

**DOI:** 10.3390/bs15060784

**Published:** 2025-06-06

**Authors:** Georgina Rosell-Bellot, Eva Izquierdo-Sotorrío, Ana Huertes-del Arco, María Rueda-Extremera, María Elena Brenlla

**Affiliations:** 1 Facultad de Ciencias de la Salud y la Educación, Universidad a Distancia de Madrid, 28010 Madrid, Spainana.huertes@udima.es (A.H.-d.A.); maria.rueda@udima.es (M.R.-E.); mariaelena.brenlla@udima.es (M.E.B.); 2 Facultad de Psicología, Universidad Nacional de Educación a Distancia, 28040 Madrid, Spain

**Keywords:** adolescence, attachment, child abuse, Complex Post-Traumatic Stress Disorder (C-PTSD), focal psychotherapy, vicarious gender-based violence

## Abstract

This study aims to illustrate the impact of accumulated traumatic experiences in adolescence and to evaluate the potential of brief focal psychotherapy (BFP) as a treatment approach for complex trauma. We present the case of a 14-year-old boy who experienced vicarious gender-based violence, child abuse, early maternal separation without alternative secure attachment figures, and forced sudden migration. The patient exhibited symptoms consistent with post-traumatic stress disorder (PTSD) and complex trauma. The culturally sensitive intervention, delivered at a public child and adolescent mental health center, consisted of twenty weekly individual sessions of 45 min each, complemented by three 45 min psychoeducation sessions with the caregiver. The assessment was conducted using a multitrait and multi-informant approach, systematically gathering information across multiple domains of functioning (emotional–behavioral, physical, cognitive, self-perception, and relational) and from different sources (the adolescent, his mother, and the clinician) through clinical interviews, projective techniques, and parental feedback. The primary therapeutic focus was the establishment of a secure therapeutic alliance to facilitate emotional exploration and trauma processing. Following treatment, the patient demonstrated significant improvements in emotional regulation, family relationships, and school performance, as measured by both self-report and parental observations. This case highlights the potential of BFP in addressing complex trauma in adolescents, particularly during a developmental stage marked by increased vulnerability to the effects of chronic trauma exposure. The findings suggest that BFP can effectively reduce both acute symptomatology and broader psychosocial consequences associated with prolonged and cumulative trauma. Further research, particularly controlled studies and longitudinal follow-ups, is needed to refine and optimize the use of BFP by mental health professionals working with adolescents affected by complex trauma.

## 1. Introduction

Traumatic life experiences take many forms, all of which can affect mental health in childhood and adolescence. Gender-based violence, child abuse, early separation from parents without the availability of other secure attachment figures, and migration have all been identified as risk factors for the development of post-traumatic stress disorder (PTSD) and complex trauma (Cárdenas Medina & Gutiérrez Espalza, 2004; Maercker et al., 2022). According to the World Health Organization (2021), one in three women experiences gender-based violence at some point in her life, and, in most cases, it is inflicted by male partners. This violence also affects the victims’ children, who are exposed to and witness the aggression, which constitutes vicarious violence (Sepúlveda, 2006). Studies indicate that, in most cases of child abuse, the aggressors perpetrate multiple types of abuse (physical and psychological) over a long period of time and are usually people from the child’s close family environment (Fañanás, 2021). Both the exposure of a child to the aggression suffered by their mother and the child abuse perpetrated against them directly have serious physical and psychopathological consequences for the child and their future. Children who experience such violence frequently suffer emotional and behavioral problems such as anxiety, depression, episodes of dissociation, aggressiveness, disruptive behaviors, relationship difficulties, somatic complaints, and other symptoms of post-traumatic stress (Ordóñez & González, 2012; Valencia, 2016). The prevalence of such symptoms is five times higher among victims of child abuse and/or vicarious gender-based violence than in the general population (Alcántara et al., 2013), and there is an impact in terms of their short- and long-term physical and psychological consequences (World Health Organization, 2022) and on school performance due to effects on social and cognitive abilities (J. M. Moreno, 2004).

Another potentially harmful experience is early separation from the primary attachment figure. Such separation and the inaccessibility of secure attachment figures in childhood can lead to significant psychological stress for the child and long-term repercussions for their mental health, representing a traumatic event in the person’s life (Garelli & Montuori, 1997; Moneta, 2014). If separation from the mother in infancy is accompanied by additional experiences with extremely neglectful attachment figures, the probability of developing a severe form of insecure attachment increases. The child does not experience the provision of a secure base and, consequently, faces great difficulties in emotional, cognitive, and social exploration (Simó, 2003). These relational experiences may cause the child to experience fear and a loss of trust towards the attachment figure and, subsequently, in relationships with other people (Moneta, 2014).

Another potentially harmful life experience to consider is migration, a process that may involve accumulated grief due to elements such as separation from family, culture, and/or group of belonging (Mezzatesta, 2016). When migration is accompanied by relevant psychosocial stressors, it can become a traumatic experience that impacts on the person’s mental health (Sánchez, 2020), prompting symptoms such as depressive symptomatology (sadness, crying, guilt, and suicidal ideation), anxious symptomatology (tension, nervousness, recurrent worries, irritability, and insomnia), somatizations (headaches and fatigue), and confusional symptomatology (failures of memory, lapses in attention, and feeling lost) (Achotegui, 2008).

The symptomatology developed in response to exposure to traumatic situations can include PTSD, which the DSM-5 defines as characterized by intrusions; the avoidance of situations associated with the traumatic event; changes in cognition and mood; and a significant alteration in the alertness and reactivity system, often causing irritable behavior and outbursts of rage and recklessness, among other possible manifestations (American Psychological Association, 2013). However, the examples of traumatic events which can cause such symptoms provided in the DSM-5 do not include vicarious violence, the experience of early separation from attachment figures, or forced migration. The term “complex trauma” aims to define a category that includes this diversity of life situations that can also be traumatizing, thereby accounting for the cumulative effects of experiencing several such situations, especially in childhood and adolescence (Nieto & López, 2016).

To meet the definition of complex trauma, traumatic events must be either sustained over a long period of time, causing damage and symptomatology to become chronic, or be suffered at multiple points in life, causing damage to accumulate (Nieto & López, 2016). Complex trauma in children and adolescents who have experienced violence, abuse, and post-traumatic reactions is associated with a specific symptomatology that can be organized into distinct domains. The emotional and behavioral domain includes difficulties in the regulation and dysregulation of emotions and impulses, somatizations, and patterns of intergenerational violence. The cognitive domain encompasses altered attention and awareness, as well as a system of meanings characterized by profound hopelessness toward people, the world, and the individual’s own future, accompanied by feelings of unhappiness and incomprehension. Within the self-perception domain, individuals often experience feelings of guilt and responsibility, which shape a negative self-image. Finally, the relational domain involves the perception of the abuser marked by acceptance and dependence, alongside difficulties in relationships with others that are characterized by distrust, vulnerability, and a pervasive sense of danger (López, 2008).

In addressing complex trauma, brief focal psychotherapy (BFP) with a psychodynamic orientation has obtained good results and is indicated for the treatment of symptomatology in child victims of abuse (Galán, 2010) and adolescents who, in addition to abuse, have suffered other traumatic experiences such as complex family contexts and migration (Mezzatesta, 2016). Evidence shows that the benefits of this treatment are lasting and not transitory, since they favor internal resources and capacities (Shedler, 2010), making them effective interventions for the child and adolescent population (Montserrat et al., 2015).

This type of intervention helps the individual to understand and cope with emotional conflict, enhancing the development of their personal resources to improve their quality of life (Rodríguez, 2021). Treatments with this orientation also aim to help the patient understand the unconscious aspects of their symptoms, enhancing their insight through the therapeutic relationship. Techniques such as free association, floating attention, mentalization, and the relational phenomena of transference and countertransference are used for the intervention objectives (Sala Morell & Equipo de Psicoterapeutas de la Red de Salud Mental, 2016). These techniques have proven to be effective in children and adolescents with a history of trauma (Bateman & Fonagy, 2016).

BFP adapts classical psychoanalysis into a brief, time-limited treatment that centers on a specific focus, defined by the conflict core involving anxieties and defense mechanisms underlying pathological functioning (Sala Morell & Equipo de Psicoterapeutas de la Red de Salud Mental, 2016). This focus is developed from a working hypothesis tailored to each patient, clarifying and connecting the various experiential and behavioral phenomena identified while also recognizing healthy and preserved aspects that can serve as mobilizing factors in treatment (Sala Morell & Equipo de Psicoterapeutas de la Red de Salud Mental, 2016). Importantly, BFP integrates concepts from attachment theory and psychodynamic approaches, emphasizing the therapeutic relationship as a secure base for exploring and processing trauma (Bateman & Fonagy, 2016; Mikulincer & Shaver, 2016). Unlike other trauma-focused therapies such as Trauma-Focused Cognitive Behavioral Therapy (TF-CBT) or Eye Movement Desensitization and Reprocessing (EMDR), which rely on structured protocols and symptom-reduction techniques (Cohen et al., 2017; Shapiro, 2018), BFP prioritizes the exploration of relational patterns and the co-construction of meaning within the therapeutic alliance (Bateman & Fonagy, 2016; Shedler, 2010). For adolescents, this approach is particularly beneficial, as it allows for flexible adaptation to developmental needs, identity formation, and evolving family dynamics (Steinberg, 2014). While TF-CBT and EMDR are effective in targeting specific traumatic memories and reducing acute symptoms (Cohen et al., 2017; Shapiro, 2018), BFP addresses the underlying interpersonal dynamics, cultural issues, and emotional conflicts that often persist in complex trauma (Bateman & Fonagy, 2016; Allen et al., 2008). This individualized and integrative model is especially valuable for adolescents who may struggle to articulate their experiences or engage in highly structured protocols, as it fosters a sense of safety, agency, and self-understanding (Bateman & Fonagy, 2016; Allen et al., 2008). By focusing on insight, emotional integration, and the restoration of trust in relationships, BFP not only alleviates symptoms but also promotes long-term psychological growth, resilience, and improved functioning in daily life (Shedler, 2010; Fonagy et al., 2015).

Although there is evidence of the efficacy of BFP (Georgievska-Nanevska, 2019; Montserrat et al., 2015; Shedler, 2010) in patients with a traumatic experience (Galán, 2010; Mezzatesta, 2016), there is a lack of studies demonstrating and refining its efficacy in addressing complex trauma, particularly when there is a diversity and accumulation of traumatic events (López, 2008). This gap is particularly relevant during adolescence, a developmental stage marked by profound neurobiological, psychological, and social changes that increase vulnerability to the effects of chronic trauma exposure (Patton et al., 2016; Andersen & Teicher, 2008). Complex trauma during adolescence can profoundly disrupt emotional regulation, cognitive functioning, self-perception, and interpersonal relationships, with consequences that extend into adulthood and affect mental health, academic achievement, and social adaptation (Amores-Villalba & Mateos-Mateos, 2017; Flynn et al., 2014; Teicher et al., 2016). Furthermore, psychosocial contexts such as family dynamics, peer relationships, and the community environment play a critical role in either exacerbating or buffering the impact of trauma, underscoring the need for multidimensional, cultural, and developmentally sensitive interventions for this population (Cicchetti & Toth, 2016; Ungar, 2013).

To address the identified gap in the literature, the present work explicitly poses the following research question: is brief focal psychotherapy (BFP) an effective intervention for adolescents presenting with complex trauma, including vicarious gender violence and child abuse?

Drawing on existing evidence, our working hypothesis is that BFP can effectively reduce complex trauma symptoms in adolescents across key domains, including emotional and behavioral regulation, cognitive functioning, self-perception, and relational dynamics. We further hypothesize that, by offering a focused, culturally sensitive, and adaptable therapeutic approach, BFP will address not only acute symptomatology but also the broader psychosocial consequences associated with prolonged and cumulative exposure to diverse traumatic events.

To explore this hypothesis, we present an in-depth clinical case study of a 14-year-old adolescent with a documented history of complex trauma. The objectives of this work are twofold: (1) to illustrate the psychopathological sequelae of chronic and multifaceted traumatic exposure during adolescence and (2) to evaluate the effectiveness of BFP in this clinical context.

## 2. Presentation of the Clinical Case

Juan (pseudonym) was born from a pregnancy in the context of a violent relationship. The mother was 16 years old when she became pregnant. There were no complications in delivery, lactation, feeding, or sleep. Language, motor, and relational development evolved adequately. The mother emphasized that he had very early sphincter control, at 12 months, due to the lack of sufficient resources such as diapers. He did not attend nursery school because he was cared for by his paternal aunt. His academic performance in elementary school was low.

Juan’s mother was a victim of gender-based violence (GBV), including severe physical and psychological aggression. This occurred daily and in Juan’s presence. To protect herself, the mother fled the relationship, leaving her country of origin and children, when Juan was 9 years old and his siblings were younger.

After his mother left, Juan’s father began to physically and psychologically abuse him as well. This included forcing him to keep very demanding schedules (getting up very early and going to bed late) to perform all household chores and care for his siblings. These obligations were combined with his school attendance. His father also belittled him in front of his siblings and humiliated him in front of other adults. He also blamed him for his mother’s departure.

He lived in this situation for four years. At the age of 13, his maternal grandmother arranged a trip to Spain to visit his mother. During this trip, Juan explained to his mother his father’s mistreatment of him. He was taken to a doctor for his physical injuries, and medical evidence confirmed the abuse he had reported. His mother decided that he would not return to her country of origin with his father and that she would keep the children with her in Spain. Thus, Juan experienced sudden migration.

Juan is currently living with his mother and two siblings. Although the family receives economic government assistance, the mother works long hours and has little time to care for Juan and his siblings. He is adjusting to his new household with a mother he has not seen in four years and experiences frequent conflicts with his siblings. He attends a public high school and is involved in extracurricular activities including tutoring for academic support and leisure activities. In these contexts, he presents relational and behavioral problems, including school absenteeism and conflicts with his peers.

All procedures described in this case study were conducted in accordance with the ethical standards of the institutional research committee and with the principles of the Declaration of Helsinki. Written informed consent was obtained from the adolescent’s mother, and assent was obtained from the adolescent prior to participation in the evaluation and intervention.

### Reason for Consultation

Based on a referral from their primary care center, Juan and his mother attended a consultation at a public child and adolescent mental health center, where both the evaluation and the intervention described below took place. The center routinely serves a migrant population and adopts a culturally sensitive approach. Previously, the guidance team at Juan’s high school alerted the mother to his recurrent absenteeism and his perceived lack of motivation. At the time of the intervention, the family was also receiving regular contact from social services due to their precarious economic situation.

Juan’s mother explained that she observed her son being sad, anxious, and irritable. She characterized him as unmotivated by anything and said that, consequently, he did not go to school unless she accompanied him. In addition, she mentioned that he had episodes of severe anger that he expressed with a certain aggressiveness, giving as an example an intense fight with his siblings. This tense situation at home made her feel unable to care for her children and manage their family life. Finally, she related Juan’s emotional state to the physical and psychological abuse he had experienced at the hands of his father and expressed the belief that her son needed space to be able to talk about these events.

In describing their family history, Juan’s mother disclosed that she had been a victim of violence and sexual abuse since childhood: Juan’s maternal grandfather and grandmother physically assaulted her, and she was raped by a stranger. Juan’s father had assaulted her physically and psychologically, daily, since their first month of living together, during her first teenage pregnancy (from which Juan was born), and after their children were born. The mother had suffered from depressive episodes and severe migraines. Juan’s maternal grandmother had also been a victim of abuse by her partner, the maternal grandfather, and had had recurrent depressive episodes. The only information that could be gathered from the father concerned his abuse of the mother and her child.

Juan’s medical history included severe migraines, which he had experienced since childhood. Doctors in Spain also detected a herniated disc and damaged ribs because of past physical abuse.

## 3. Materials and Methods

This work adopts a qualitative single-case study design, which allows for an in-depth exploration of the clinical presentation, therapeutic process, and outcomes in an ecological context. In line with recommendations for child assessment (Eid, 2014; Izquierdo-Sotorrío et al., 2016), a multimethod, multi-informant, and multitrait approach—implemented in a culturally sensitive manner—was used in the assessment process. The multitrait component refers to the evaluation of multiple domains of functioning (emotional–behavioral, physical, cognitive, self-perception, and relational), while the multi-informant component involved gathering information from different sources (the adolescent, his mother, and the clinician).

Both the evaluation and the therapeutic intervention were conducted by the same professional, a junior health psychologist at the center. The whole therapeutic process received regular supervision from the center’s senior health psychologist, who reviewed the clinical formulation, intervention plan, and progress in accordance with institutional protocols. This supervisory framework ensured the quality and ethical standards of the intervention.

### 3.1. Evaluation

The evaluation process was carried out in three 45 min sessions. The first session was attended by the mother, the second by the mother and Juan, and the third session by only Juan.

In psychodynamic and trauma-focused brief focal psychotherapy, clinical observation, projective techniques, and clinical judgment are often prioritized over standardized psychometric instruments. Accordingly, a semi-structured clinical interview, genogram (Yanes et al., 2002), lifeline (Guerra, 2019), and free drawing were employed as projective techniques in the assessment process. In line with current recommendations, a multimethod, multitrait, and multi-informant approach was adopted. Information was systematically gathered from multiple sources (the adolescent, his mother, and the clinician) and across several domains of functioning (emotional–behavioral, physical, cognitive, self-perception, and relational). Pre- and post-treatment assessments were mainly conducted through a culturally sensitive interview, as no psychometric instruments adapted to the patient’s cultural background were available at the center. Additionally, clinical observation and projective techniques provided valuable qualitative data to complement the information obtained from interviews and informants. Therefore, clinical observation during the interviews focused on the key domains affected by complex trauma: emotional and behavioral regulation, cognitive functioning, self-perception, and relational dynamics.

The interview served to establish first contact, explore the reason for the consultation, establish an initial rapport, and collect information about Juan’s life history and concerns (Marín, 2021). Some information regarding Juan’s history of abuse could not be collected due to the difficulty he had in talking about the aggressors and their relatives. Despite this challenge, enough relevant information was collected to establish a pattern of transgenerational family violence (Arias et al., 2017). Juan had been exposed to multiple traumatic events throughout his childhood and adolescence. From birth until the age of 9, he had witnessed daily physical and psychological violence by his father against his mother. He was then separated from his mother when he was 9 years old, leaving him in the exclusive care of his father, who subjected him to continuous physical and psychological abuse for the next four years. During this time, Juan assumed disproportionate domestic responsibilities and suffered constant humiliation, accompanied by physical aggression.

At the age of 13, Juan migrated unexpectedly to his grandmother and was reunited with his mother, whom he had not seen for four years. The resulting loss of his familiar environment and the adaptation to a new family reality were marked by tensions in living with his mother and siblings. The family was also facing economic difficulties, adding another level of stress to the context of adaptation.

At the time of this first appointment, Juan presented persistent sadness, anxiety, and anger, with frequent episodes of irritability and difficulty in controlling his emotions. He experienced intrusive memories of the mistreatment he had suffered and avoided situations or emotions that reminded him of the traumatic events. He also expressed a negative view of himself, others, and his environment and maintained a constant sense of hypervigilance. He also showed signs of demotivation, with a lack of interest in recreational and school activities, as well as conflicts in his interpersonal relationships, both in the family and social spheres.

Physically, Juan experienced severe migraine attacks and episodes of chronic fatigue. He manifested impulsivity, aggressive behaviors, and risky attitudes, such as association with conflictive groups. These difficulties had a significant impact on his daily functioning in family, school, and social environments.

The free drawing technique was used to explore Juan’s personality, functioning, significant bonds, and areas of conflict (Charras et al., 2007; Freud, 1980). This technique facilitates the analysis of problems that do not appear in explicit communication. Its application consists of asking the person to draw a picture and then proposing that they tell a story about this drawing (Charras et al., 2007). Juan drew a landscape with mountains, rivers, trees, birds flying overhead, and two people in a tent. In his story, he described a couple in love contemplating a sunset and the migration of birds. According to the manual interpretation for this projective technique (Buck, 2008), the drawing and story, which seem to represent a reality far removed from Juan’s life in the present and recent past, could indicate a defense mechanism associated with denial or affective isolation.

In summary, Juan’s clinical presentation demonstrates the pervasive impact of complex trauma across multiple domains. Emotionally and behaviorally, he showed persistent sadness, anxiety, irritability, intrusive memories, avoidance of reminders of traumatic events, demotivation, impulsivity, aggressive behaviors, and challenges in emotional regulation. Physically, he reported severe migraine attacks and episodes of chronic fatigue. Cognitively, his symptoms included hypervigilance, intrusive thoughts, the avoidance of certain topics, and difficulties in discussing aspects of his abuse. Regarding self-perception, Juan expressed a negative self-image and feelings of guilt and responsibility. Relationally, his history included exposure to family violence, separation from caregivers, tensions in family relationships, and conflicts in both family and social settings.

### 3.2. Explanatory Hypotheses and Diagnosis

The traumatic events that Juan had lived through acted as antecedents influencing the appearance of the above-mentioned problems. Witnessing his father assaulting his mother, staying behind when his mother fled the violence without him or his siblings, the childhood and psychological abuse he suffered, and the unexpected migration experience are stress and trauma factors that may have facilitated the development of psychopathology (Nieto & López, 2016).

If we analyze all these traumatizing experiences, it appears they may have prevented him from establishing attachment bonds with a secure base (López & Ramírez, 2005). Since he was born, his close figures, who were supposed to be sensitive and caring, demonstrated violence and failed to protect him, generating feelings of abandonment, guilt, and confusion, leading to disorganized insecure attachment (Muela et al., 2012). The boy had witnessed prolonged abuse against his mother, which constituted both direct and vicarious trauma (Courtois & Ford, 2012). Additionally, his migratory experience entailed cumulative stressors such as cultural displacement known to compound trauma in migrant youth (Perreira & Ornelas, 2011).

Such experiences in early relationships and attachment style influenced Juan’s internal operating models (IOMs) (González, 2022). The mental models that he internalized about himself, his relationships with others, and his environment informed his ability to understand and predict how others would act and what his behaviors, thoughts, and emotions should be (Fresno et al., 2012). These mental representations—constructed without a secure base—combine to create the perception of a hostile and threatening world against which he must defend himself alone (Lecannelier et al., 2011), prompting anger, irritability, and conflict at school and at home.

He also appeared to observe this dangerous and frightening world with heightened alertness and reactivity, causing him to startle when subjected to small stimuli, to the point of acting impulsively. Additionally, being exposed to violence in various forms and on a continuous basis may have normalized stressful situations and, therefore, triggered reckless behaviors such as joining conflictive peer groups (Lecannelier et al., 2011). At the same time, they may have decreased his motivation in the face of a hostile environment that he could do nothing to avoid.

Violent and chaotic bonds in the first years of life can increase a person’s ambivalence towards their traumatic experiences and make them question whether they were guilty or deserving of such treatment. Amidst such life circumstances, defense mechanisms of affective isolation may be activated (Bustos & Russo, 2018; Morandini, 2022) to avoid emotions to decrease suffering, maintain a relationship with the attachment figure, and ensure survival (Fresno et al., 2012). This internal mechanism results in mood disturbances and emotional regulation difficulties, leading Juan to exhibit aggressiveness and angry outbursts when he is unable to control his internal rage (Sezer & Gürtepe, 2025).

Now that he has returned to live with his mother, he is in a more protected situation, out of danger and away from his abusive father. Even so, there is still a feeling of insecurity in the maternal bond due to his early life experiences and his mother’s abandonment of him, which did not allow him to build a secure base. Thus, a disorganized attachment style and the lack of a secure base seem to explain the origin and maintenance of Juan’s current functioning, which manifests in psychological distress, relationship difficulties, and behavioral problems (Lecannelier et al., 2011). 

Juan meets all of the DSM-5 diagnostic criteria (American Psychological Association, 2013) for post-traumatic stress disorder and Confirmed Childhood Physical and Psychological Abuse (Initial Finding). In Juan, we also observe the following symptoms characteristic of complex trauma (López, 2008): (1) difficulties in emotional regulation that make him act impulsively and struggle to control his intense rage; (2) flat affect and a lack of expression of feelings when he talks about his traumatic experiences; (3) alterations in self-perception with feelings of guilt for the aggression he experienced and for having distanced himself from his father; (4) alterations in his perception of his abuser, including missing him and having good memories of him; (5) relationship difficulties, including feelings of distrust towards others; (6) somatizations including severe migraines, fatigue, and excessive sleep; and (7) feelings of discouragement, sadness, and a lack of interest and motivation towards life.

### 3.3. Treatment

The treatment objectives focused on addressing, from a culturally sensitive perspective, Juan’s emotional, relational, and behavioral needs to promote his well-being. This approach considered the specific impact of his migration experience and cultural background, recognizing how forced migration, adaptation to a new environment, and the challenges of acculturation shaped both his trauma and his coping strategies.

The overall goal was to facilitate the processing of traumatic events to reduce his symptoms, providing a secure base through a therapeutic bond that strengthened his confidence, emotional well-being, and relational capacities. Treatment also sought to enhance his capacity for self-awareness so that he could better understand and connect with his emotions, perceptions, and behaviors as well as the traumatic events he has experienced. A further aim was to promote personal resources that would allow him to identify, express, and manage his emotions, thus reducing psychological discomfort and behavioral problems.

As for the specific objectives, the treatment sought to reduce the alteration in mood, which manifested in sadness, anxiety, anger, and persistent anger and irritability, and to improve emotional regulation and increase self-control. The aim was to reduce Juan’s tendency to distance himself emotionally and avoid feelings associated with his trauma, as well as to reduce the frequency and intensity of intrusive memories related to the traumatic experiences. Another objective was to modify his negative perceptions of himself, his relationships, and the world while also addressing his feelings of guilt and his constant sense of being in danger. The aim was to identify and reduce emotional ambivalence to traumatic events while working to reduce hypervigilance and heightened reactivity. A related goal was to reduce his aggressive behaviors, outbursts of anger, and reckless attitudes, as well as to address somatic manifestations, such as migraine attacks. Finally, the treatment aimed to increase his motivation to participate in daily activities, reduce school absenteeism, and foster a more positive and harmonious family life.

On the other hand, the sessions with the mother were aimed at facilitating her understanding of her child’s needs and functioning, helping her to develop resources that would allow her to better meet these needs, and offering practical strategies to manage family conflicts and improve coexistence at home.

#### Treatment Plan

The planned treatment, which was agreed with both Juan and his mother, was brief focal psychotherapy (BFP) with a psychodynamic orientation. The BFP involved 20 individual weekly sessions of 45 min each. Three 45 min psychoeducation sessions with Juan’s mother were also scheduled at the beginning, middle, and end of Juan’s treatment, i.e., quarterly. In this treatment, the explanatory hypothesis described above was taken into account to establish the focus on the conflictive core that influenced Juan’s global functioning, leading to a central effort to construct a therapeutic relationship that served as a safe base to feel greater tranquility, security, and protection, thereby modifying the internal work model to reduce his emotional, social, and behavioral symptomatology. Providing a secure base would allow him to have a different relational experience from the aggressive and neglectful one he had experienced thus far. Another consideration was that if he had the capacity to feel secure in a relationship, he would have space for listening and containment, facilitating the emotional exploration and processing of his traumatic experiences (Rodríguez, 2021). Therapeutic formulation considered the intersectionality of trauma and migration, prioritizing cultural responsiveness. The therapist adopted a stance of cultural humility and explicitly explored the client’s cultural identity, expectations, and worldview (Hook et al., 2016). The specific objectives proposed with the mother, meanwhile, included being able to offer more security to Juan by facilitating better knowledge of her child’s functioning and needs and strengthening resources to respond to challenges and family conflicts at home.

Table 1 shows the schedule, phases, techniques, and objectives of the intervention sessions with Juan and the psychoeducation sessions with the mother. The effectiveness of the intervention was assessed using a multitrait, multi-informant approach. Specifically, information was systematically gathered throughout the therapeutic sessions from multiple sources, including clinical observation, self-reported symptoms by the adolescent, parental feedback collected during psychoeducation sessions, and information about school attendance and behavior (multi-informant). This process allowed for a comprehensive evaluation of changes across several domains of functioning—emotional, behavioral, cognitive, physical, and relational (multitrait)—over the course of the intervention.

To better understand the techniques used in this BFP, it is useful to explain that at the beginning of each session, Juan was offered a listening space so that he could talk about his week. In this way, free association was encouraged (Georgievska-Nanevska, 2019). The therapist was attentive to what Juan chose to discuss and used these topics to serve the therapeutic goals of that session (Braier, 2016). During the conversation, mentalization was used by asking reflective questions, pointing, rephrasing, and validating thoughts and feelings (Sala Morell & Equipo de Psicoterapeutas de la Red de Salud Mental, 2016). This approach was intended to help Juan to better understand himself, his emotions, his behaviors, and the people around him and to comprehend the possible relationship between his functioning and his traumatic experiences (Ballespí, 2017).

The transference and countertransference processes were considered in each session (Sala Morell & Equipo de Psicoterapeutas de la Red de Salud Mental, 2016). In most of the sessions, Juan expressed feelings of severe anxiety and concern regarding the conflicts caused by his discomfort and aggressiveness. He projected the need for protection and containment by someone externally in the face of his loss of control and suffering. Identifying these needs helped to guide the treatment, supporting Juan to develop resources that would allow him to live with a greater sense of calm. Meanwhile, the psychoeducation sessions increased the mother’s resources for responding to her son’s needs. To further illustrate how these dynamics were addressed within the therapeutic process, and to offer a more comprehensive account of how the secure-base therapeutic alliance—the central focus of this intervention—was established, Table 2 outlines the content and objectives of the corresponding sessions.

## 4. Results

### 4.1. Emotional, Behavioral, and Physical Functioning

The BFP process produced significant improvements in the adolescent’s emotional regulation and behavioral functioning. The adolescent demonstrated greater awareness of the intensity of his emotions, as shown by statements such as “I get angry about everything, even for a little look someone else may give me”. He was able to verbalize his emotions regarding traumatic experiences, for example, being able to cry when recounting an incident of aggression. He improved his ability to identify and express his emotions in conflict situations, stating, “It makes me so angry that I can’t control it, it can’t stay like this, I’ll punch him”. He also began to develop more adaptive strategies for emotional management, such as recognizing the disproportionality of some impulsive reactions, as in, “I threw the cell phone and broke it, I went too far, if I really could have told him to stop”.

These developments led to increased emotional stability and self-control. His emotional flattening and mood disturbances decreased, along with an overall improvement in emotional well-being. Additionally, his expression of feelings increased, and there was a reduction in reactivity and impulsivity, which went from occurring daily to rarely. There was a reduction in aggressive behaviors, outbursts of rage, and somatizations. The adolescent’s improved emotional management also contributed to a reduction in persistent anger and irritability, promoting greater well-being and better adaptation to his environment.

Physically, there was a marked decrease in symptoms such as migraines and chronic fatigue from monthly to occasionally.

### 4.2. Cognitive Functioning and Self-Perception

There was a significant increase in the adolescent’s capacity for insight and self-reflection. He began to question his own behaviors and recognize their origins, as shown by statements such as “I don’t know why I always get into trouble now”. He was able to identify the relationship between his traumatic experiences and his current emotions and behaviors, which was illustrated by his saying, “I am angry with everyone because of my father”, and further evidenced when he noted, “I learned this from him, he told me that if I did not defend myself from the threats of others, he would beat me up later”. He also began to recognize and elaborate on ambivalences and altered perceptions, as demonstrated by his observations: “My father tells me that it is my fault that I have not gone back to him and that my mother has a partner, but I have nothing to do with it” and “I have experienced things that I should not have experienced”. Through this process, he managed to create a more compassionate and coherent narrative of his traumatic experiences, a shift captured in his reflection: “Before I was angry, but now I think I understand. My mother left because otherwise she would be dead. If she hadn’t left, I wouldn’t be here now”.

These cognitive and reflective advances contributed to a reduction in persistent anger, irritability, intrusive memories, negative beliefs about himself, others, and the world, ambivalences regarding his father and traumatic events, feelings of guilt, and reckless behaviors. There was also a reduction in hypervigilance, intrusive memories, and avoidance of traumatic memories from daily to weekly.

Juan expressed a negative view of himself, marked by feelings of guilt and responsibility. This negative self-image was reinforced by his experiences of humiliation and disproportionate domestic responsibilities during the years he lived exclusively with his father. Through the therapeutic process, he began to question and deconstruct the sense of responsibility imposed by his father.

### 4.3. Relational Functioning

A key therapeutic objective was to offer a secure base through the therapeutic bond. Over the course of the intervention, the adolescent demonstrated a clear reduction in insecurity and distrust within the therapeutic relationship. For example, after a period of missed appointments, he began arriving punctually to every session, reflecting a reduction in resistance and increased engagement.

He developed a growing sense of safety and trust in the therapeutic bond, as he expressed the following: “You always remember everything we talked about”. This sense of security enabled him to express and process traumatic experiences that he had previously been unable to share with anyone else, as illustrated by his following statement: “I hadn’t been able to tell anyone about this yet”.

Importantly, this experience of a secure base was not limited to the therapy setting. The adolescent was able to internalize and generalize this relational security to other areas of his life. This was evident in the formation of new friendships at school and in his increased willingness to share current concerns with his mother.

As a result of these relational changes, there was a reduction in negative beliefs about himself, others, and the world. He also experienced a diminished sense of danger, hostility, and constant threat. Hypervigilance and relational difficulties also decreased, as did the frequency of interpersonal conflicts both at home and at school (from weekly to monthly).

Another positive outcome was a significant increase in his motivation to improve and plan for his future. His school attendance improved, with the adolescent attending every day, although he continued to face challenges in meeting academic expectations. In the final session, the free drawing technique was used again (Charras et al., 2007). The adolescent drew a scene almost identical to the initial assessment, but this time described the figures not as a romantic couple, but as a mother protecting her son, watching over him as he rested on the grass, reflecting his perception of safety and protection at that moment.

### 4.4. Caregiver Psychoeducation and Family Impact

The psychoeducation sessions with the adolescent’s mother also led to developments with the potential to improve the emotional well-being of the adolescent and his family. The mother shared her own traumatic experiences she had suffered as a child and the mistreatment she experienced from Juan’s father, expressing the hope that these experiences would not hinder her ability to care for her son. For example, the mother also struggled with her anger and punished the adolescent when he exhibited aggressive behaviors with his siblings because she was not able to understand how he could act this way after his own experience of abuse. The psychoeducation sessions provided the mother with a more comprehensive understanding of her son and helped her learn strategies to care for and set limits for him in a more sensitive and functional way. On the other hand, she expressed concern for her other children, who were beginning to show maladaptive functioning.

### 4.5. Follow-Up

The adolescent presented encouraging results, but due to the complexity and diversity of his traumatic experiences, behavioral problems continued to be observed that hindered his functioning. According to the center protocols, a follow-up plan (Muñoz, 2003) was established, consisting of monthly sessions with the adolescent during the first year after therapeutic discharge, complemented by bimonthly interviews with his mother. The aim was to sustain progress, identify potential risk indicators at an early stage, and reinforce the coping strategies developed throughout the therapeutic process.

## 5. Discussion

This paper presents a psychodynamically oriented intervention using BFP for the treatment of an adolescent with complex trauma and post-traumatic stress symptomatology at a public child and adolescent mental health center. As the results obtained show, BFP appears to be an effective intervention for Juan, consistent with the evidence collected in the literature indicating the efficacy of psychodynamic treatment in children and adolescents (Galán, 2010; Georgievska-Nanevska, 2019; Mezzatesta, 2016; Montserrat et al., 2015; Shedler, 2010).

The diversity of traumatic experiences suffered by Juan and its consequent symptomatology transcends the category of PTSD, making it relevant to consider the concept of complex trauma when making a diagnosis (López, 2008). In the approach to this case, it was important to acknowledge the term “complex trauma” to carry out an intervention that would account for all the particularities and complexities of Juan. In cases such as this, the diversity of Juan’s traumatic experiences must be fully understood to understand the functioning of the person and adapt the treatment to their needs.

BFP has proven adaptable to the demands of a public child and adolescent mental health service such as the one in which this intervention was carried out. Its brief format makes it possible to treat patients at a time when there is a high demand for care. In addition, the establishment of a focus makes it possible to target concrete and specific problems with the treatment, thereby making it easier to accomplish the therapeutic objectives and to signal the end of the intervention (García & Vélez, 1990).

The focus on providing a secure base as an intervention based on attachment theory allowed Juan to explore his current circumstances, reflect on the connections between his past and present, and better understand his emotions and behaviors (Rodríguez, 2021). This focus allowed him to develop a perception of security and confidence in an attachment that enabled him to use anxieties and defensiveness to establish a mode of functioning that reduced his emotional, behavioral, physical, and relationship problems (Aguilar, 2019). The change in the adolescent’s drawing at the end of treatment, from a couple in love to a mother protecting her son, may be interpreted as an increased sense of security within his family relationships (A. Moreno, 2019). This symbolic shift suggests that the therapeutic process contributed to a reconfiguration of his attachment representations.

Addressing the phenomenon of vicarious gender-based violence (VGBV) played a fundamental role in the effectiveness of the intervention. Given that exposure to gender-based violence in the family or social environment can generate profound psychological repercussions in adolescents (Ordóñez & González, 2012; Valencia, 2016), recognizing and integrating this dimension into the therapeutic approach was essential. A focus on VGBV allowed for a deeper understanding of Juan’s emotional and relational patterns, as well as the internalization of traumatic experiences that influenced their functioning. By incorporating this perspective, BFP was adapted to address not only the immediate post-traumatic symptomatology but also the broader psychosocial impact, enhancing the intervention’s capacity to foster resilience and emotional regulation. Psychodynamic interventions for children and adolescents who have experienced trauma emphasize the importance of establishing a secure therapeutic relationship to facilitate the processing of traumatic memories and the development of mentalization capacities, thereby promoting emotional regulation and resilience (Bateman & Fonagy, 2016).

Additionally, the therapeutic alliance, strengthened through culturally sensitive practices, proved to be a key facilitator of change (Hook et al., 2016). These outcomes are consistent with research on the efficacy of culturally adapted trauma interventions for migrant youth (Sirin & Rogers-Sirin, 2015), suggesting that addressing the combined impact of interpersonal trauma and forced migration within a culturally responsive framework can enhance therapeutic engagement and symptom reduction.

The case illustrates the importance of considering the full complexity of trauma presentations in adolescents, especially when multiple adversities such as VGBV and migration are present. Recognizing and integrating these dimensions into the intervention allows for a more comprehensive and effective therapeutic process. Furthermore, the adaptability and focus of BFP demonstrates its suitability for public mental health services, where efficiency and clear therapeutic objectives are essential (Sala Morell & Equipo de Psicoterapeutas de la Red de Salud Mental, 2016).

Important limitations in the evaluation and treatment of this case warrant explicit consideration, particularly considering the assessment and intervention strategies previously described. Firstly, although comprehensive clinical information was obtained through culturally sensitive interviews and projective techniques, the absence of standardized psychometric instruments—such as the Trauma Symptom Checklist for Children (TSCC) or the Child Behavior Checklist (CBCL)—represents a significant methodological constraint. While these tools were not available at the treatment center, their use would have been both feasible and appropriate given the patient’s age, enabling a more objective and quantifiable assessment of symptom severity and behavioral disturbances associated with complex trauma. The inclusion of standardized measures would have allowed the systematic tracking of symptom changes over time and the application of rigorous statistical analyses (e.g., non-overlapping data indices) to determine the clinical significance of therapeutic effects (J. Sanz et al., 2015). Consequently, the lack of such instruments limits the empirical robustness and generalizability of the reported treatment outcomes.

Secondly, although the assessment process incorporated several projective techniques and clinical observation, the evaluation could have been further enriched using additional projective tools to explore the patient’s personality structure, affective defenses, and intrapsychic functioning in greater depth (Siquier de Ocampo et al., 2003). The omission of these tools restricts the comprehensiveness of the clinical formulation and may have limited the depth of understanding regarding the patient’s internal world.

Thirdly, the treatment plan focused primarily on individual intervention through brief focal psychotherapy (BFP) and did not include systemic family-level approaches. Given the psychological difficulties and relational dysfunction identified within the family system, the absence of family psychotherapy may have constrained the therapeutic impact. Incorporating family interventions could have addressed emotional and behavioral issues affecting multiple family members, potentially enhancing overall efficacy and improving family dynamics (L. Sanz et al., 2009; Vázquez & Vázquez, 2023). Without this systemic perspective, it is difficult to disentangle the specific effects of BFP from broader familial and environmental influences.

Also, potential confounding factors, including the psychological background of the patient’s mother and other family members, may have influenced the observed outcomes. The inherent limitations of a single-case design preclude the ability to unequivocally attribute changes solely to the intervention, as contextual variables may also play a role. Future research employing controlled designs and larger samples is necessary to clarify the specific contribution of BFP in the treatment of adolescents with complex trauma.

In summary, this case highlights the importance of providing a secure therapeutic environment and culturally sensitive interventions for adolescents who have experienced complex trauma. Improvements were observed across multiple domains of functioning: emotional and behavioral regulation, physical symptoms, cognitive processing, self-perception, and relational dynamics. These changes were assessed using a multimethod approach, following a multitrait (multiple domains) and multi-informant (adolescent, caregiver, and clinician) framework. The observed progress underscores the potential of brief focal psychotherapy to address both acute symptoms and broader psychosocial challenges in this population. Future research should continue to refine these interventions and explore their applicability in diverse clinical settings.

## 6. Conclusions

This case study presents an adolescent who experienced vicarious gender-based violence and child abuse, resulting in complex trauma. The intervention, brief focal psychotherapy with a psychodynamic orientation and caregiver psychoeducation, contributed to meaningful improvements across emotional, behavioral, and physical functioning; cognitive functioning and self-perception; and relational functioning. These outcomes were systematically evaluated using a multitrait and multi-informant approach, integrating information from the adolescent, his mother, and the clinician. These findings highlight the clinical value of individualized, developmentally appropriate, and culturally sensitive approaches, particularly those grounded in brief psychodynamic frameworks, for adolescents with histories of interpersonal violence and abuse. Further research is needed to confirm these findings and inform best practices for clinical interventions with this vulnerable population.

## Figures and Tables

**Table 1:** Scheduling, phases, techniques, and objectives of the BFP treatment sessions.

Session No.	Intervention	Attendees	Technique	Session Objectives
1	Evaluation	Mother	Interview	First visit Reason for consultation explained by the mother
2	Evaluation	Mother and patient	Lifeline	Collect information on psychobiography Explore current symptomatology
3	Evaluation	Patient	Free drawing	Identify Juan’s symptomatology and functioning
1B	Psychoeducation	Mother	Interview	Establish the setting Facilitate knowledge of Juan’s needs
1–10	BFP	Patient	Transference and countertransference	Set the setting Build a secure therapeutic bond, reducing resistance and consolidating trust in the relationship Promote the capacity for insight
2B	Psychoeducation	Mother	Interview	Follow up on treatment with Juan and update on Juan’s current situation Facilitate knowledge of Juan’s needs Enhance resources to respond to patient’s needs
11–20	BFP	Patient	Free drawing	Build a secure therapeutic bond to promote the internalization of the relational experience Promote the capacity for insight Help patient develop internal resources for emotional management Work on treatment closure and the generalization of results
3B	Psychoeducation	Mother	Interview	Facilitate knowledge of Juan’s needs Enhance resources to respond to Juan’s needs Close the intervention and provide a follow-up proposal

**Table 2:** Treatment sessions with BFP: building a secure-base therapeutic alliance.

Session No.	Objectives and Session Content
1–4	Establish the therapeutic setting. Identify resistance and ambivalence. Example: the patient says, “I don’t like talking about my stuff,” but arrives very punctually to all sessions. Highlight and communicate resistance and ambivalence to the patient. Address the needs necessary to establish safety and trust in the therapeutic relationship.
5–8	Build the secure-base therapeutic alliance through a space of attentive listening, understanding, and emotional containment.
9–10	Identify behaviors that serve to verify the safety of the relationship. Example: the patient missed two sessions, which were later rescheduled. Identify beliefs about relationships with others. Example: the patient expressed that they expected the therapist to be angry about their absences. Modify these beliefs within the relational experience by offering a space of listening, understanding, and emotional containment.
11–14	Consolidate the secure-base therapeutic relationship through a space of listening, understanding, and emotional containment.
15–20	Prepare for termination and prevent anxiety related to separation: revisit the therapeutic setting, including the number of sessions completed and remaining, and recall reflections developed during the sessions to strengthen the patient’s internal resources. Consolidate the learning from the relational experience and generalize the outcomes: example: being able to feel secure in relationships with others in their environment.

Note: some objectives are maintained throughout the entire course of treatment, although they are listed under specific sessions for the purpose of better illustrating the intervention’s progression.

## Data Availability

The datasets presented in this article are not readily available because they contain sensitive information from a clinical case involving a minor. Requests to access the datasets should be directed to the corresponding author.

## Abbreviations

The following abbreviations are used in this manuscript:

BFP	Brief Focal Psychotherapy
PTSD	Post-Traumatic Stress Disorder
C-PTSD	Complex Post-Traumatic Stress Disorder
DSM-5	Diagnostic and Statistical Manual of Mental Disorders, Fifth Edition
GBV	Gender-Based Violence
